# Risk of Adverse Reproductive Outcomes Associated with Proximity to Municipal Solid Waste Incinerators with High Dioxin Emission Levels in Japan

**DOI:** 10.2188/jea.14.83

**Published:** 2005-03-18

**Authors:** Toshiro Tango, Toshiharu Fujita, Takeo Tanihata, Masumi Minowa, Yuriko Doi, Noriko Kato, Shoichi Kunikane, Iwao Uchiyama, Masaru Tanaka, Tetsunojo Uehata

**Affiliations:** 1 Department of Technology Assessment and Biostatistics, National Institute of Public Health.; 2 Department of Epidemiology, National Institute of Public Health.; 3 Department of Health Promotion and Research, National Institute of Public Health.; 4 Department of Water Supply Engineering, National Institute of Public Health.; 5 Department of Environmental Engineering, Graduate School of Engineering, Kyoto University.; 6 Graduate School of Natural Science and Technology, Okayama University.; 7 Faculty of Humanities, Seitoku University.

**Keywords:** birth weight, infant mortality, fetal death, congenital, hereditary, and neonatal diseases and abnormalities, sex ratio

## Abstract

BACKGROUND: Great public concern about health effects of dioxins emitted from municipal solid waste incinerators has increased in Japan. This paper investigates the association of adverse reproductive outcomes with maternal residential proximity to municipal solid waste incinerators.

METHODS: The association of adverse reproductive outcomes with mothers living within 10 km from 63 municipal solid waste incinerators with high dioxin emission levels (above 80 ng international toxic equivalents TEQ/m^3^) in Japan was examined. The numbers of observed cases were compared with the expected numbers calculated from national rates adjusted regionally. Observed/expected ratios were tested for decline in risk or peak-decline in risk with distance up to 10 km.

RESULTS: In the study area within 10 km from the 63 municipal solid waste incinerators in 1997-1998, 225,215 live births, 3,387 fetal deaths, and 835 infant deaths were confirmed. None of the reproductive outcomes studied here showed statistically significant excess within 2 km from the incinerators. However, a statistically significant peak-decline in risk with distance from the incinerators up to 10 km was found for infant deaths (p=0.023) and infant deaths with all congenital malformations combined (p=0.047), where a “peak” is detected around 1-2 km.

CONCLUSION: Our study shows a peak-decline in risk with distance from the municipal solid waste incinerators for infant deaths and infant deaths with all congenital malformations combined. However, due to the lack of detailed exposure information to dioxins around the incinerators, the observed trend in risk should be interpreted cautiously and there is a need for further investigation to accumulate good evidence regarding the reproductive health effects of waste incinerator exposure.

There has been great public concern that adverse reproductive health effects may be associated with dioxins in emission gases from municipal solid waste (MSW) incinerators. Dioxin, the name loosely assigned to a class of chemicals referring to 210 different PCDD (polychlorinated dibenzo-*p*-dioxins) and PCDF (dibenzofurans) congeners, has been shown to be a carcinogen, a teratogen, and a reproductive toxicant in animals.^1^ In Japan, it is estimated that more than 90 percent of exposure to dioxins in daily life comes from food, primarily fish, in the general human population.^1^ However, it seems to be natural to consider that individuals living around the MSW incinerators are exposed to dioxins coming from emission gases more than those living far from the incinerators. Up to the present, it remains to be determined whether individuals living near the MSW incinerators are exposed to dioxins at doses sufficient to produce adverse reproductive health effects.^2^

So far, studies of the adverse reproductive effects of dioxins in humans are limited.^3^^-^^17^ Of these, studies of maternal environmental exposures to dioxins are only a few. In 1976, a chemical plant explosion had occurred in Seveso, Italy, and the highest community exposures to dioxins were documented. The high exposure to dioxins in parents from Seveso were shown to be linked to a statistically significant lowered male/female sex ratio in their offspring, which persisted for years after exposure.^14^^-^^15^ However, the sex ratio was not altered among offspring of women exposed to 1978-1979 Taiwan Yucheng incidents^16^ that produced high levels of dioxins similar to those of Seveso accident. A study of birth defects in mothers with potential exposure to dioxins showed an apparent increased risk for infant, fetal and perinatal death, low birth weight, and several subcategories of birth defects, but none of these were statistically significant.^17^

In April 11, 1997 in Japan, the press released that the Ministry of Health and Welfare of Japan revealed the results of the survey of the MSW incinerators. Of the 1,150 MSW incinerators surveyed, dioxin emissions from 72 were above 80 ng international toxic equivalents TEQ/m^3^, which was set as the standard for taking urgent countermeasure against dioxin emissions from the MSW incinerators by Environmental Agency of Japan. This finding strongly motivated us to investigate whether or not adverse reproductive outcomes cluster in the vicinity of these MSW incinerators with high dioxin emission levels.

The purpose of this study is to examine the adverse reproductive health effects associated with maternal residential proximity to these incinerators with high dioxin emission levels in Japan.

## METHODS

### Selection of incinerators

Selection of the MSW incinerators in our study was based upon the emission levels of MSW incinerators surveyed by the Ministry of Health and Welfare of Japan. The survey report published in April 1997 included only one measurement per MSW incinerator and no repetitive measurements from which we can estimate the average and the variability of emission levels for each incinerator. However, by considering that (1) the operation condition of incinerators is usually controlled within a pre-defined constant range and day-to-day variation of emission levels seems to be not so large, and that (2) the Ministry of Health and Welfare of Japan has urged an urgent countermeasure against dioxin emissions for the MSW incinerators based upon these measurements, we decided to use this report to select MSW incinerators.

**Figure 1.**
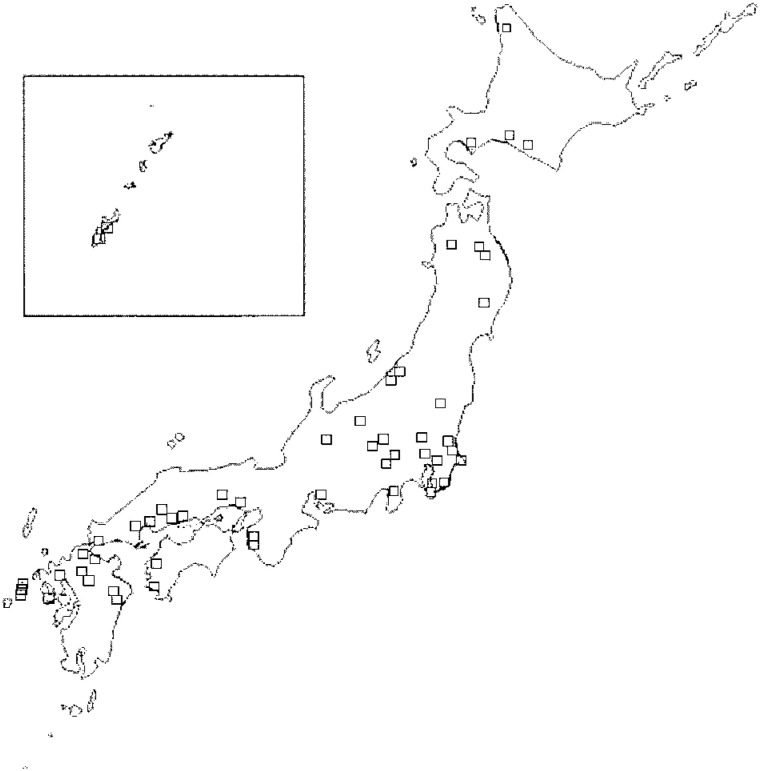
Selected 63 municipal solid waste incinerator sites in Japan. Included in the box are Amami-Okinawa region consisting of several islands.

As MSW incinerators with dioxin emission levels above 80 ng TEQ/m^3^ at the time of survey in 1997, 72 MSW incinerators were chosen at outset to be candidate for this study. However, we excluded nine MSW incinerators that we could not obtain the exact address information at the beginning of our study. As a result, 63 MSW incinerators were selected for this study (Figure 1).

### Study area, study region and study population

In our study, two similar terms “study area” and “study region” are used differently. The “study area” was defined as circles of radius 10 km from the MSW incinerators. To identify the study population that was defined as all live births, fetal deaths, and infant deaths occurred in the study area during a 2 year period of 1997 and 1998, we selected all the municipalities (“city”, “ward”, “town” and “village”) that were located within, or overlapped with the study area. The group of municipalities selected for each MSW incinerator was defined as the “study region” which is wider than the “study area” and is used as an adjustment factor to calculate the expected number of cases taking into account the regional difference of standardized mortality or incidence ratios. A total of 451 municipalities were selected.

To identify all the study population members, we first used vital statistics records of live births, fetal deaths, and deaths. In the study region, we confirmed 489,154 live births, 7,242 fetal deaths, and 1,796 infant deaths during 1997-1998. Although these vital statistics records include all the study population members, they do not contain detailed street addresses because of privacy protection. To compute the “distance” of individual address from the corresponding MSW incinerator, we copied the street addresses from the birth, fetal death, and death certificate forms by hand-search. Then, using a coordinate matching program of ArcView GIS,^18^ a software of Geographical Information System, these street addresses were converted into latitude and longitude coordinates (gecoding). Inadequate addresses, or those for which no matching addresses were found were considered missing and were excluded from all the analyses. These address-matching processes successfully geocoded 92.2% of live births, 92.9% of fetal deaths, and 91.5% of infant deaths. Finally, in the “study region”, we confirmed 451,041 live births, 6,728 fetal deaths, and 1,644 infant deaths. In the “study area” within 10 km from the MSW incinerators, we confirmed 225,215 live births, 3,387 fetal deaths, and 835 infant deaths.

### Cases and national rates

The selected reproductive outcomes studied were female live births (male/female sex ratio at birth), low birth weight (weighing less than 2,500g), very low birth weight (weighing less than 1,500g), infant deaths (under one year of age), infant deaths due to congenital malformations, neonatal deaths (under four weeks of age), neonatal deaths due to congenital malformations, early neonatal deaths (under one week of age), early neonatal deaths due to congenital malformations, spontaneous fetal deaths (non-induced deaths before the complete expulsion or extraction from the mother after the 12th week of gestation), and spontaneous fetal deaths with congenital malformations. Because spontaneous fetal deaths do not include non-induced deaths before the 12th week of gestation, our study might have an incomplete accounting of reproductive defects. It will not be unreasonable to assume, however, that most observable anomalies due to exposure to dioxins, if any, could be detected after the 12th week since the first trimester is usually considered to be truly the exposure period of interest in terms of congenital defects and there are some incubation period between exposure and manifestation of toxicity effect.

To calculate the expected number of cases, we obtained national rates based on all live births, fetal deaths, and infant deaths occurred in the study area during 1997-1998 in Japan. The national rate for female births was simply based on the total female live births divided by the total live births delivered in Japan during 1997-1998, and was calculated to be 1,178,580 / (1,178,580 + 1,241,062) = 0.487 (male/female sex ratio = 1.053). However, national rates for other outcomes were stratified by potential confounding factors available from the corresponding vital statistics records: maternal age, gestational age, birth weight, total previous deliveries, past experience of fetal deaths, and type of paternal occupation.

### Statistical analysis

Each study area was divided into ten sub-areas (called “zone”) delimited by ten circles of radii of 1, 2, …., 10 km. The statistical analysis of the association between maternal proximity to the incinerators and adverse reproductive outcomes was primarily based upon the observed (O) and expected (E) numbers of cases. This procedure is very similar to that described in details elsewhere.^19^ For descriptive purposes, the observed and expected numbers of cases, O/E ratios and their 95% confidence intervals (CIs; calculated assuming a Poisson distribution) are reported for the study area (0-10 km) and for an area close to the MSW incinerator, arbitrary selected to be (0-2 km). As formal tests, we used Stone’s unconditional test^20^ and Tango’s conditional test^21^ for decline in risk (O/E ratio) with distance from the incinerator. Motivated by the dioxin levels in soils measured in the vicinity of MSW incinerators^22^ where a peak of concentration was found around 2 km from MSW incinerators, we also applied Tango’s conditional test^21^ for “peak-decline” in risk with distance where the location of “peak” was restricted to be one of three zones, 0-1 km (decline model), 1-2 km, and 2-3 km.

The hypothesis testing was for all the MSW incinerators combined, as have been recommended in the literatures.^23^^-^^25^ For the unconditional test, the null hypothesis is the same for all the incinerators, i.e., constant O/E ratio of 1.0 (regional average) in the all ten zones. The data can therefore be aggregated across sources, within zones, and a single test performed assuming that there is no interaction between incinerators. For the conditional test, on the other hand, the null hypotheses are unique to each incinerator. The tests therefore have to be performed separately for each incinerator and the results combined. P-values of these unconditional and conditional tests were calculated using 9,999 Monte Carlo simulations, and the nominal statistical significance levels were taken to be p=0.05. Combining the p-values, *p_i_*’s, from each MSW incinerator was based on the weighted inverse normal method:^26^ a statistics $\overset{63}{\underset{\text{i}=1}{\Sigma }}w_{\text{i}}\Phi^{-1}(p_{\text{i}})$ is distributed with standard Normal distribution with mean 0 and variance 1, where Φ(.) denotes the standard Normal distribution function with mean 0 and variance 1, ${\text{w}}_{\text{i}}=\sqrt{\left\{O_{\text{i}}/(\Sigma O_{\text{j}})\right\}}$ and *O_i_* denotes the observed number of cases in the *i*-th study area. When *O*i = 0, then *w*_i_Φ^-1^(*p*_i_) was defined as zero.

### Ethical consideration

Although our study was based on individual vital statistics records of live births, fetal deaths, and deaths, our analyses are based upon the observed number of cases in each zone. Therefore, it is unlikely that there arises some ethical issues from our study.

## RESULTS

Tables 1 to 6 show, for each of adverse reproductive outcomes and all the MSW incinerators combined, O/E ratios, cumulative O/E ratios, 95% CIs of cumulative O/E by distance from MSW incinerators and one-tailed p-value of Stone’s unconditional test for decline in O/E ratio with distance from the incinerators. None of adverse reproductive outcomes showed significant excess for all the zones. Apparently, a decline in risk with distance from the MSW incinerators was observed for female live births and spontaneous fetal deaths with congenital malformations, and a peak-decline trend was observed for infant deaths, infant deaths due to congenital malformations, and early neonatal deaths. However, all the Stone’s unconditional tests for decline in risk with distance were not statistically significant. 

**Table 1.** Female live births near 63 municipal solid waste incinerators by distance of residence from the incinerator, in 1997-1998 in Japan.

Distance* (km)	No.		O/E	Cumulative O/E (95% CI)	Stone’s unconditional p-value

	Observed (O)	Expected (E)			
[0,1)	1114	1071.21	1.04	1.04 (0.98-1.10)	0.075
[1,2)	4402	4521.30	0.97	0.99 (0.96-1.01)	
[2,3)	7174	7270.55	0.99	0.99 (0.97-1.00)	
[3,4)	8348	8299.98	1.01	0.99 (0.98-1.01)	
[4,5)	9629	9563.02	1.01	1.00 (0.99-1.01)	
[5,6)	12614	12644.65	1.00	1.00 (0.99-1.01)	
[6,7)	15109	14829.80	1.02	1.00 (1.00-1.01)	
[7,8)	16244	15974.14	1.02	1.01 (1.00-1.01)	
[8,9)	17135	16831.67	1.02	1.01 (1.00-1.02)	
[9,10)	18275	18366.32	1.00	1.01 (1.00-1.01)	

*: [*a,b*) denotes the interval from *a* to *b*, which includes *a* but does not include *b*. Distance between home and closest municipal solid waste incinerator.

CI: confidence interval.

**Table 2.** Low birth weights and very low birth weights near 63 municipal solid waste incinerators by distance of residence from the incinerator, in 1997-1998 in Japan.

Distance* (km)	No.		O/E	Cumulative O/E (95% CI)	Stone’s unconditional p-value

	Observed (O)	Expected (E)			
	Low birth weight ( < 2500 g )				

[0,1)	170	166.83	1.02	1.02 (0.87-1.18)	0.786
[1,2)	758	754.35	1.01	1.01 (0.94-1.07)	
[2,3)	1142	1198.86	0.95	0.98 (0.94-1.02)	
[3,4)	1359	1366.12	1.00	0.98 (0.95-1.02)	
[4,5)	1610	1616.91	1.00	0.99 (0.96-1.02)	
[5,6)	2175	2156.60	1.01	0.99 (0.97-1.02)	
[6,7)	2554	2532.56	1.01	1.00 (0.98-1.02)	
[7,8)	2692	2693.27	1.00	1.00 (0.98-1.02)	
[8,9)	2735	2758.08	0.99	1.00 (0.98-1.01)	
[9,10)	2972	3013.73	0.99	1.00 (0.98-1.01)	

	Very low birth weight ( < 1500 g )				

[0,1)	14	13.72	1.02	1.02 (0.56-1.71)	0.594
[1,2)	58	59.83	0.97	0.98 (0.77-1.23)	
[2,3)	86	89.28	0.96	0.97 (0.83-1.13)	
[3,4)	109	104.46	1.04	1.00 (0.88-1.13)	
[4,5)	138	123.56	1.12	1.04 (0.94-1.14)	
[5,6)	171	179.94	0.95	1.01 (0.93-1.10)	
[6,7)	183	184.24	0.99	1.01 (0.94-1.08)	
[7,8)	205	206.33	0.99	1.00 (0.94-1.07)	
[8,9)	192	201.41	0.95	0.99 (0.94-1.05)	
[9,10)	238	251.37	0.95	0.99 (0.94-1.04)	

*: [*a,b*) denotes the interval from *a* to *b*, which includes *a* but does not include *b*. Distance between home and closest municipal solid waste incinerator.

CI: confidence interval.

**Table 3.** Infent deaths and infant deaths due to congenital malfornation near 63 municipal solid waste incinerators by distance of residence from the incinerator, in 1997-1998 in Japan.

Distance* (km)	No.			O/E	Cumulative O/E (95% CI)	Stone’s unconditional p-value

	Observed (O)		Expected (E)			
Infant deaths						

[0,1)	7	7.75		0.90	0.90 (0.36-1.86)	0.298
[1,2)	43	30.81		1.40	1.30 (0.96-1.71)	
[2,3)	54	52.53		1.03	1.14 (0.93-1.38)	
[3,4)	43	64.44		0.67	0.95 (0.80-1.11)	
[4,5)	63	73.13		0.86	0.92 (0.80-1.05)	
[5,6)	111	102.08		1.09	0.97 (0.87-1.08)	
[6,7)	121	113.15		1.07	1.00 (0.91-1.09)	
[7,8)	134	125.33		1.07	1.01 (0.93-1.10)	
[8,9)	129	122.35		1.05	1.02 (0.95-1.10)	
[9,10)	130	141.42		0.92	1.00 (0.94-1.07)	

Infant deaths ( all congenital malformations combined )						

[0,1)	2	2.66		0.75	0.75 (0.09-2.72)	0.411
[1,2)	17	11.48		1.48	1.34 (0.81-2.10)	
[2,3)	19	18.97		1.00	1.15 (0.81-1.58)	
[3,4)	14	21.95		0.64	0.94 (0.71-1.24)	
[4,5)	27	25.94		1.04	0.98 (0.77-1.22)	
[5,6)	41	34.46		1.19	1.04 (0.86-1.24)	
[6,7)	53	40.89		1.30	1.11 (0.95-1.28)	
[7,8)	48	43.46		1.10	1.11 (0.97-1.26)	
[8,9)	38	43.09		0.88	1.07 (0.94-1.20)	
[9,10)	51	48.85		1.04	1.06 (0.95-1.19)	

*: [*a,b*) denotes the interval from *a* to *b*, which includes *a* but does not include *b*. Distance between home and closest municipal solid waste incinerator.

CI: confidence interval.

**Table 4.** Neonatal deaths under four weeks and neonatal deaths due to congenital malfornation near 63 municipal solid waste incinerators by distance of residence from the incinerator, in 1997-1998 in Japan.

Distance* (km)	No.		O/E	Cumulative O/E (95% CI)	Stone’s unconditional p-value

	Observed (O)	Expected (E)			
Neonatal deaths					

[0,1)	4	4.42	0.91	0.91 (0.25-2.32)	0.523
[1,2)	21	16.71	1.26	1.18 (0.77-1.75)	
[2,3)	31	28.77	1.08	1.12 (0.85-1.46)	
[3,4)	23	36.13	0.64	0.92 (0.73-1.15)	
[4,5)	38	39.48	0.96	0.93 (0.77-1.12)	
[5,6)	60	56.21	1.07	0.97 (0.84-1.13)	
[6,7)	68	60.64	1.12	1.01 (0.89-1.15)	
[7,8)	78	69.43	1.12	1.04 (0.93-1.16)	
[8,9)	76	65.85	1.15	1.06 (0.96-1.17)	
[9,10)	72	79.82	0.90	1.03 (0.94-1.13)	

Neonatal deaths ( all congenital malformations combined )					

[0,1)	2	1.67	1.20	1.20 (0.15-4.32)	0.629
[1,2)	9	6.93	1.30	1.28 (0.64-2.29)	
[2,3)	12	11.88	1.01	1.12 (0.71-1.69)	
[3,4)	9	12.94	0.70	0.96 (0.66-1.35)	
[4,5)	20	15.18	1.32	1.07 (0.80-1.40)	
[5,6)	20	20.58	0.97	1.04 (0.81-1.31)	
[6,7)	30	24.67	1.22	1.09 (0.89-1.32)	
[7,8)	31	26.58	1.17	1.10 (0.93-1.31)	
[8,9)	22	25.64	0.86	1.06 (0.90-1.24)	
[9,10)	31	30.21	1.03	1.06 (0.91-1.22)	

*: [*a,b*) denotes the interval from *a* to *b*, which includes *a* but does not include *b*. Distance between home and closest municipal solid waste incinerator.

CI: confidence interval.

**Table 5.** Early neonatal deaths under seven days and early neonatal deaths due to congenital malfornation near 63 municipal solid waste incinerators by distance of residence from the incinerator, in 1997-1998 in Japan.

Distance* (km)	No.		O/E	Cumulative O/E (95% CI)	Stone’s unconditional p-value

	Observed (O)	Expected (E)			
Early neonatal deaths					

[0,1)	3	3.12	0.96	0.96 (0.20-2.81)	0.337
[1,2)	16	11.78	1.36	1.28 (0.77-1.99)	
[2,3)	19	19.41	0.98	1.11 (0.78-1.52)	
[3,4)	12	23.61	0.51	0.86 (0.64-1.14)	
[4,5)	22	26.39	0.83	0.85 (0.67-1.08)	
[5,6)	44	37.72	1.17	0.95 (0.79-1.14)	
[6,7)	47	40.36	1.17	1.00 (0.86-1.17)	
[7,8)	51	46.72	1.09	1.02 (0.89-1.17)	
[8,9)	56	43.39	1.29	1.07 (0.95-1.21)	
[9,10)	44	53.59	0.82	1.03 (0.92-1.15)	

Early neonataldeaths ( all congenital malformations combined )					

[0,1)	1	1.01	0.99	0.99 (0.03-5.51)	0.619
[1,2)	5	4.30	1.16	1.13 (0.42-2.46)	
[2,3)	6	7.42	0.81	0.94 (0.49-1.65)	
[3,4)	4	7.87	0.51	0.78 (0.44-1.26)	
[4,5)	12	9.39	1.28	0.93 (0.62-1.35)	
[5,6)	14	12.78	1.10	0.98 (0.71-1.33)	
[6,7)	19	14.65	1.30	1.06 (0.81-1.37)	
[7,8)	24	16.63	1.44	1.15 (0.92-1.42)	
[8,9)	15	15.13	0.99	1.12 (0.91-1.36)	
[9,10)	21	18.65	1.13	1.12 (0.93-1.34)	

*: [*a,b*) denotes the interval from *a* to *b*, which includes *a* but does not include *b*. Distance between home and closest municipal solid waste incinerator.

CI: confidence interval.

**Table 6.** Spontaneous fetal deaths and spontaneous fetal deaths due to congenital malfornation near 63 municipal solid waste incinerators by distance of residence from the incinerator, in 1997-1998 in Japan.

Distance* (km)	No.		O/E	Cumulative O/E (95% CI)	Stone’s unconditional p-value

	Observed (O)	Expected (E)			
Spontaneous fetal deaths					

[0,1)	26	31.72	0.82	0.82 (0.54-1.20)	0.971
[1,2)	131	125.58	1.04	1.00 (0.85-1.17)	
[2,3)	222	235.90	0.94	0.96 (0.87-1.07)	
[3,4)	253	242.72	1.04	0.99 (0.92-1.07)	
[4,5)	308	303.83	1.01	1.00 (0.94-1.07)	
[5,6)	337	384.17	0.88	0.97 (0.91-1.02)	
[6,7)	492	463.04	1.06	0.99 (0.94-1.04)	
[7,8)	460	490.58	0.94	0.98 (0.94-1.02)	
[8,9)	558	528.70	1.06	0.99 (0.96-1.03)	
[9,10)	593	556.34	1.07	1.01 (0.97-1.04)	

Spontaneous fetal deaths ( all congenital malformations combined )					

[0,1)	4	2.29	1.75	1.75 (0.48-4.48)	0.552
[1,2)	9	6.45	1.40	1.49 (0.79-2.54)	
[2,3)	13	13.62	0.96	1.16 (0.76-1.70)	
[3,4)	15	12.46	1.20	1.18 (0.85-1.60)	
[4,5)	15	17.50	0.86	1.07 (0.81-1.39)	
[5,6)	19	22.88	0.83	1.00 (0.79-1.25)	
[6,7)	27	28.62	0.94	0.98 (0.80-1.19)	
[7,8)	26	29.39	0.89	0.96 (0.80-1.14)	
[8,9)	32	31.83	1.01	0.97 (0.83-1.13)	
[9,10)	42	31.16	1.35	1.03 (0.89-1.18)	

*: [*a,b*) denotes the interval from *a* to *b*, which includes *a* but does not include *b*. Distance between home and closest municipal solid waste incinerator.

CI: confidence interval.

Regarding the conditional test based on the weighted inverse normal method, there were no significant declines in risk with distance for all adverse reproductive outcomes. However, a significant peak-decline in risk with distance was found for infant deaths (p= 0.023), and infant deaths with all congenital malformations combined (p= 0.047) (Table 7).

**Table 7.** Results of Tango’s conditional tests for the municipal solid waste incinerators combined, by reproductive outcome.

Reproductive outcomes	The number of study areas combined*	Tango’s conditional p-value	

		decline	peak-decline
Female live births	63	0.880	0.948
Low birth weight (<2500g )	63	0.836	0.911
Very low birth weight (< 1500g )	56	0.551	0.746
Infant deaths	47	0.122	0.023
Infant deaths due to congenital malformations	42	0.138	0.047
Neonatal deaths	40	0.363	0.199
Neonatal deaths due to congenital malformations	35	0.245	0.204
Early neonatal deaths	38	0.488	0.308
Early neonatal deaths due to congenital malformations	31	0.505	0.518
Spontaneous fetal deaths	61	0.941	0.897
Spontaneous fetal deaths due to congenital malformations	35	0.449	0.502

* The number of combined study areas with non-zero observed cases : O _i_ > 0

## DISCUSSION

Our study is a Japan’s first-ever large-scale nationwide study to examine the adverse reproductive health effects associated with maternal residential proximity to the 63 MSW incinerators with high dioxin emission levels in Japan. Quite a similar study, in its design and statistical analyses, of health effects related to MSW incineration among general population has been performed in Great Britain,^27^^-^^28^ where cancer incidence near the MSW incinerators has been investigated.

Our main finding is that there was a small but statistically significant “peak-decline” in risk with distance from the MSW incinerators up to 10 km for both infant deaths and infant deaths with congenital malformations. None of previous studies have detected a cluster of infant deaths and infant deaths with congenital malformation around MSW incinerators. However, careful observation of the O/E ratio by distance from the MSW incinerators shown in Tables 1 to 6 indicated that there was a “hollow” in the zone 3-4 km in contrast with a peak in the zone 1-2 km for infant deaths and infant deaths due to congenital malformation. The similar observation was found for neonatal deaths and early neonatal deaths. One possible speculation is that these fluctuations were observed as a result of simple random variation of O/E ratio over distance. However, nobody may have the right answer without further information. That is why we introduced some statistical tests for detecting clusters of disease around the MSW incinerators. Another statistical issue we should consider to some extent is that our significant findings detected for infant deaths and infant deaths with congenital malformations were obtained in one of the three tests performed for each of reproductive outcomes. In the strict sense, we will face the problem of multiple testing which increases the nominal significance level. If we apply Bonferroni procedure, all our significant findings will be altered to be nonsignificant. Because we applied different tests for detecting different types of disease clusters, however, we considered it inappropriate to adjust for multiple testing.

Needless to say, the statistical significant evidence of association in this study cannot demonstrate causality because of several limitations in the data and methods. A major difficulty is the lack of detailed exposure information to dioxin around the incinerators. Factors such as chimney stack height, abatement equipment, and the direction of prevailing wind could influence the distribution of dioxin levels around the incinerators. Further, it is impossible to measure maternal blood or cord blood, or breast milk levels of dioxin of all the mothers delivered. In the absence of such exposure information, even a simple model of decline in risk with distance, equal in all directions, has been used to examine environmental excess risks around putative sources.^23^^-^^25^^,^^27^^-^^28^ Regarding the blood levels of dioxins around MSW incinerators, the investigator group of Ibaraki prefecture has conducted a well-designed study whose primary purpose is to examine the association of the resident’s blood levels of dioxins with the distance from the Shiratori MSW incinerator in Ryugasaki, Ibaraki.^29^ At the design stage, 120 residents were selected so as to adjust sex, age, and distance from the incinerator. As a result, a locally weighted smoothing method revealed a weak decline in dioxin levels with distance. As a part of our study, we have already examined the dioxin levels in soil samples within the circle of radius 10 km from two randomly selected MSW incinerators.^22^ The dioxin levels in both areas, although median levels are different with each other, suggest a similar weak non-significant decline in dioxin levels with distance from the incinerator. Interestingly, the highest concentrations were found at 1.9 km in one site and at 2 km in the other site, indicating that a peak of concentration was found around 2 km from the MSW incinerator in each site. Of course, these data cannot be used to describe the general pattern of other study areas due to difference between incinerators, however, the observed peak-decline in risk with distance for infant deaths and infant deaths with all congenital malformations combined might reflect most of the distribution of dioxin levels around the MSW incinerators.

Regarding the reliability of outcome data, the total number of live births, fetal deaths, and infant deaths are fairly accurate because the notifying system of birth and death in Japan is fairly complete^30^ according to the Family Registration Raw. The number of deaths due to congenital malformations, on the other hand, is relatively less accurate because there could be misclassifications of the type of diagnoses to some extent.

The observed association of infant deaths and those with congenital malformations with distance from the MSW incinerators may have different explanations due to socioeconomic status of household that we could not include in the analysis. Because of chosen or imposed circumstances, people living near MSW incinerators could be subject to social disadvantages.^31^ Especially, it is well documented that the socioeconomic status of women has a predictive value for low birth weight.^32^ In our study, however, estimated O/E ratios for both low birth weight and very low birth weight have shown no associations with the distance from MSW incinerators, indicating indirectly that socioeconomic confounding was unlikely for infant deaths and other related deaths.

Although several potential confounding factors were controlled by computing stratified national rates, information on other relevant variables, such as smoking and alcohol history, maternal and paternal occupational exposures to dioxin, and nutritional status, were not available from the birth, fetal, and death certificates. Smoking status during pregnancy, especially late pregnancy period, could be an important risk factor for growth retardation, low birth weight and infant morbidity.^33^ The impact of emissions from active industrial facilities operating and from farmers’ burning waste vegetables near MSW incinerators is another factor that could not be adequately addressed in this study. However, impact of these factors on the general population in causing adverse reproductive outcomes is far from certain and it is unlikely that a “peak” of “peak-decline” trend observed at around 1-2 km from the MSW incinerators could be mainly explained by these different emissions.

We assumed that a risk of exposure existed if a woman lived in proximity to MSW incinerators at the time she delivered. However, we had no information on each mother’s actual exposure to dioxin and her duration of exposure before delivery. Furthermore, maternal address listed on the birth, fetal death, and death certificates may not be an accurate measure of dioxin exposure. The residence at delivery for the mother may not be the residence of the mother during her first trimester which was considered as the period of the greatest concern with respect to chemical exposure. Furthermore, no information was available regarding relative mobility of pregnant women around the MSW incinerators, e.g., migration of mothers away from incinerators and migration toward incinerators of unexposed mothers. Misclassification of mother’s residential exposure due to these imprecise information would be nondifferential and would usually lead to bias toward the null. Finally, it should be noted that we cannot deny the existence of any environmental risk factors related to the peak observed around 1-2 km other than dioxins but that identification of such factors is beyond our present study.

In conclusion, our study suggests a small but statistically significant peak-decline in risk with distance from the MSW incinerators for infant deaths and infant deaths due to congenital malformations. We have no detailed exposure information to dioxins around the incinerators studied here, however, statistical significant findings cannot directly indicate causality. Furthermore, because our study is the first nationwide epidemiologic study to examine the adverse reproductive health effects associated with maternal residential proximity to the incinerators, there seems to be no other comparable epidemiologic evidence. Therefore, further studies on incineration and human health are needed to corroborate our results.
